# Genome-Wide Investigation and Functional Analysis Reveal That *CsKCS3* and *CsKCS18* Are Required for Tea Cuticle Wax Formation

**DOI:** 10.3390/foods12102011

**Published:** 2023-05-16

**Authors:** Lilai Mo, Xinzhuan Yao, Hu Tang, Yan Li, Yujie Jiao, Yumei He, Yihe Jiang, Shiyu Tian, Litang Lu

**Affiliations:** 1 College of Tea Science, The Key Laboratory of Plant Resources Conservation and Germplasm Innovation in the Mountainous Region (Ministry of Education), Guizhou University, Guiyang 550025, China; 2 Department of Agricultural Engineering, Guizhou Vocational College of Agriculture, Qingzhen 551400, China

**Keywords:** cuticle wax, *KCS* gene family, tea plant (*Camellia sinensis*), wax biosynthesis

## Abstract

Cuticular wax is a complex mixture of very long-chain fatty acids (VLCFAs) and their derivatives that constitute a natural barrier against biotic and abiotic stresses on the aerial surface of terrestrial plants. In tea plants, leaf cuticular wax also contributes to the unique flavor and quality of tea products. However, the mechanism of wax formation in tea cuticles is still unclear. The cuticular wax content of 108 germplasms (Niaowang species) was investigated in this study. The transcriptome analysis of germplasms with high, medium, and low cuticular wax content revealed that the expression levels of *CsKCS3* and *CsKCS18* were strongly associated with the high content of cuticular wax in leaves. Hence, silencing *CsKCS3* and *CsKCS18* using virus-induced gene silencing (VIGS) inhibited the synthesis of cuticular wax and caffeine in tea leaves, indicating that expression of these genes is necessary for the synthesis of cuticular wax in tea leaves. The findings contribute to a better understanding of the molecular mechanism of cuticular wax formation in tea leaves. The study also revealed new candidate target genes for further improving tea quality and flavor and cultivating high-stress-resistant tea germplasms.

## 1. Introduction

The waxy layer of the plant epidermis is an important part of the plant cuticle, which is the key to resisting the external environment and protecting plants from abiotic and biotic stress. Cuticular waxes are embedded in cutin (intracuticular waxes) or form crystalline surface coatings on stems, leaves, flowers, and fruit surfaces (epicuticular waxes). These are composed of very long-chain fatty acids (VLCFAs) and their derivatives, which are lipids with chains longer than 20 carbons. Plants regulate VLCFAs to generate several types of wax crystals in the outer layer of the leaf, resulting in wax layers of varying thickness depending on environmental factors such as temperature and humidity [1,2].

In plants, VLCFA biosynthesis is controlled by a condensing enzyme: β-ketoacyl-CoA synthase (*KCS*) [3,4,5,6]. The *KCS* proteins have two conserved domain architectures 3-oxoacyl-[acyl-carrier protein (ACP)] synthase III C domain and a Type III polyketide synthase-like domain [7,8,9,10]. The *KCS* gene is responsible for synthesizing wax precursors and is a key rate-limiting enzyme for VLCFAs extension [11]. The expression level and substrate preference of *KCS* determine the final chain length and composition of VLCFAs [5,12,13,14,15,16]. For instance, the *KCS* from *Lunaria annua* has been introduced into Camelina (*Camelina sativa*) to produce nervonic acid (C24:1) [5,17]. *AtKCS1* and *AtKCS11* have broad substrate specificity for detecting saturated and monounsaturated C_16_-C_24_ acyl-CoA, while *AtKCS17* is specific for saturated fatty acyl-CoA substrates [18]. The fatty acid elongation 1 (*FAE1*) gene from *Brassica napus* is overexpressed in the rapeseed to increase the content of erucic acid (C22:1), whilst its silencing lowers the contents of VLCFAs [19,20,21].

*KCS* gene families regulate the content and composition of epidermal wax, actively participate in physiological and biochemical responses at all stages of plant growth and development, and provide plant stress tolerance. A total of 21 *KCS* family genes have been identified in *Arabidopsis thaliana* with varying tissue distribution characteristics and expression levels, and five of them have been shown to encode *KCS* and produce corresponding VLCFAs [8]. During salt stress, the fatty acid elongation pathway as well as the keratin and wax synthesis pathways were activated in rice. Almost all wax synthesis pathways genes such as *OsKCS*, *OsKCR,* and *OsCER* were significantly upregulated in the leaves [6,14]. Similarly, in sorghum, salt treatment induced high expression of *SbKCS14*, *SbKCS16,* and *SbKCS18*, while significantly downregulating *SbKCS8* [22].

The most popular beverage in the world is tea (*Camellia sinensis*), a perennial woody plant grown in temperate and tropical regions. Environmental stressors such as low temperature [23], drought [24] pathogen infection [25], and insect pests [26] have an adverse impact on tea yield and quality. Tea cuticle wax acts as a barrier to protect tea plants from extreme weather and biotic stresses by enhancing the cuticle’s water barrier properties or adjusting the wax layer thickness and structure [27,28]. Numerous biotic and abiotic stresses suffered by tea plants result in a significant reduction in tea yield and quality, as well as huge economic losses to local agriculture. Consequently, understanding the mechanism of plant stress resistance and investigating the associated genes have significant theoretical and practical implications. While the *KCS* genes play an essential role in the synthesis of waxes and VLCFAs, relatively little is known about the *KCS* gene family of the tea plant.

Recently, the transcript levels of *CsFATB*, *CsLACS*, *CsKCS,* and other genes of the wax synthesis pathway were found to be elevated under low-temperature stress, leading to an increase in the total leaf wax. Particularly, *CsKCS2* and *CsKCS9* were highly expressed in germplasms that were tolerant to low-temperature stress [29]. Caffeine is the predominant component of tea skin wax [30] and one of the key metabolites that determine the flavor of tea [31]. Thus, tea cuticle wax is not only an important component of tea resistance to stress but also influences tea flavor quality.

The present work comprehensively investigated the leaf cuticular wax content and the transcriptome profiles of different tea germplasms and screened *CsKCS* family members as candidate regulatory genes for controlling the cuticular wax formation in tea leaves. The physicochemical properties, subcellular localization, gene chromosomal localization, phylogenetic relationship, gene structure, protein-conserved motif order, cis-acting elements and collinear relationships, tissue specificity, and environmental response expression patterns of the *CsKCS* family members were analyzed. The VIGS strategy was implemented to validate the indispensable regulatory role of *CsKCS3* and *CsKCS18* in the formation of cuticular wax in tea leaves. This work enriches the understanding of the regulatory network of cuticle wax formation in tea leaves and provides pertinent insights for further research.

## 2. Results

### 2.1. Correlation Analysis between Cuticle Wax and Expression Profile

**Figure 1:**
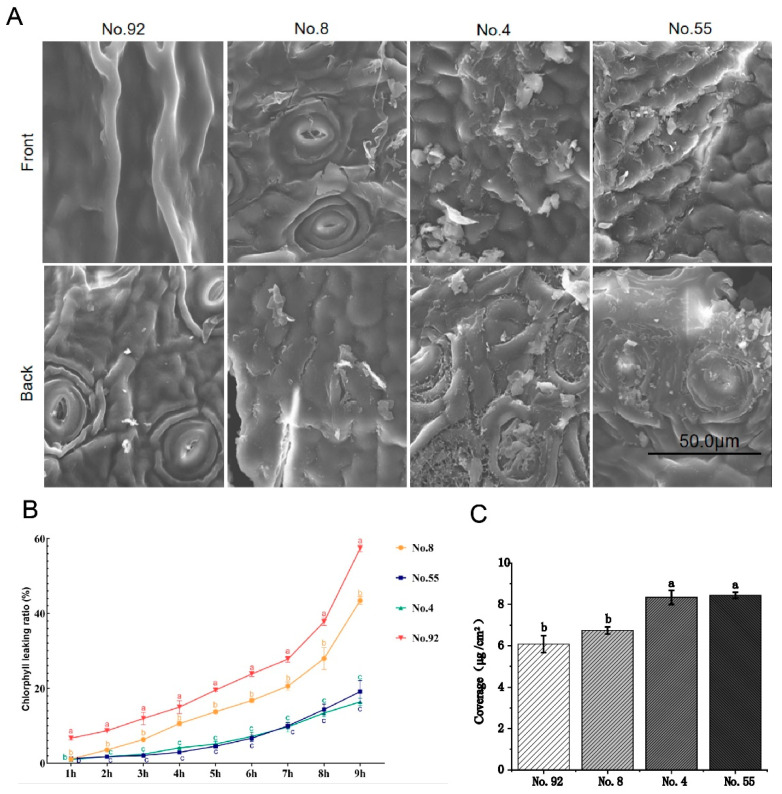
(**A**) The phenotype of wax crystals on the surface of the Niaowang species tea plant, was visualized by electron microscopy. The bar indicated 50.0 µm. (**B**) Chlorophyll leakage rate of tea leaves (different Niaowang species; No. 4, 8, 55, 92) with different cuticle contents. (**C**) Cuticular wax content in leaves of No. 4, No. 8, No. 55, and No. 92 Niaowang species. Three technical and biological replicates were used for each data point. Data were presented as means ± Sd (*N* = 3). Different letter in histograms denotes significant difference (*p* < 0.05).

To understand the cuticle wax content of different tea germplasms, scanning electron microscopy was used to observe the cuticle wax of 108 tea germplasms, sourced from Guiding Yunwu. The cuticle wax content of some germplasms differed significantly (Table 1), and four representative germplasms were selected for further study. The tea leaves from No. 4 and No. 55 Niaowang species contain high content of cuticular wax crystals, followed by medium content in No. 8 Niaowang species, and the least cuticular wax crystals in No. 92 Niaowang species (Figure 1A).

To assess the accumulation of cuticular wax in the leaves of No. 4, No. 8, No. 55, and No. 92 Niaowang species, the chlorophyll leaching rate of tea leaves was measured (Figure 1B). The chlorophyll leaching rate of No. 92 Niaowang species was the highest, followed by No. 8 Niaowang species, and the chlorophyll leaching rate of No. 4 and No. 55 Niaowang species was the lowest.

The contents of cuticular wax in leaves of No. 4, No. 8, No. 55, and No. 92 Niaowang species were determined. The results showed that the contents of cuticular wax in leaves of No. 4 and No. 55 Niaowang species were the highest, the content of cuticular wax in leaves of No. 4 Niaowang species was medium, and the content of cuticular wax in leaves of No. 92 Niaowang species was the lowest (Figure 1C, Table S1).

**Figure 2:**
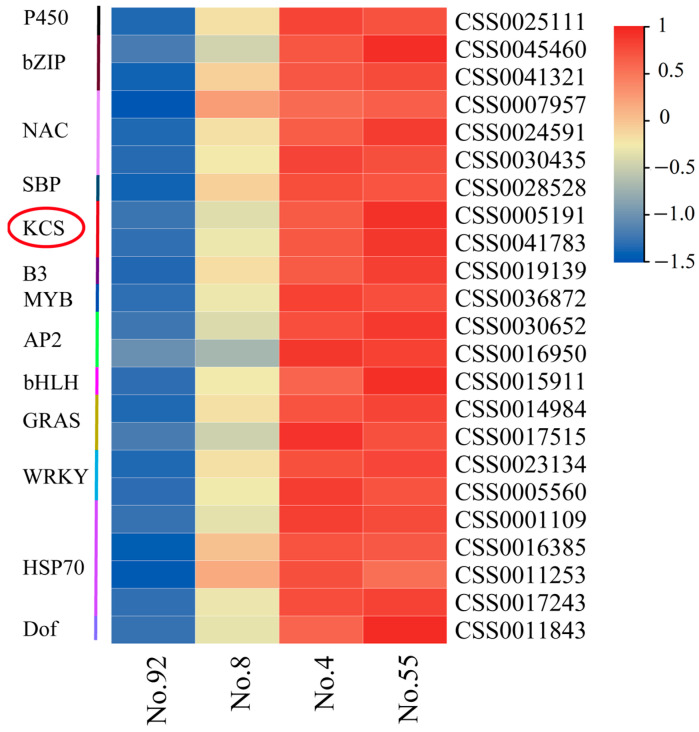
Heatmap of hypothetical gene expression changes involved in wax synthesis in the cuticle of tea leaves. The numbers (No. 4, 8, 55, 92) correspond to respective Niaowang species. The gene family of *CsKCS* is indicated with a red circle.

The RNA-seq transcriptome profiles of mature leaves of four tea germplasms (No. 4, No. 8, No. 55, No. 92) were further compared to identify the key genes affecting the synthesis of cuticular wax in tea leaves. The first 23 upregulated genes (Table S2) with the highest fold change in expression among the four tea germplasms and significant differences in leaf cuticular wax content were extracted from the transcriptome data. These genes belong to 13 gene families. Though the *KCS* gene family has been reported to play an important role in the formation of cuticle wax [5,11,12,13,14,15], their regulatory functions in the formation of cuticular wax in tea leaves have not been studied so far. Figure 2 shows that the expression levels of the two candidates’ KCS genes (gene ID: CSS0005191 and CSS0041783) were higher in tea germplasms with high leaf cuticle wax content (No. 4 and No. 55 Niaowang species), followed by those in tea germplasms with medium leaf cuticle wax content (No. 8 Niaowang species), and the expression level was the lowest in tea germplasms with low leaf cuticle wax content (No. 92 Niaowang species). Therefore, the phylogenetic relationship, gene and protein structure characteristics, expression patterns, and functional analysis of *KCS* family members were probed further to clarify their regulatory roles in cuticular wax synthesis in tea leaves.

### 2.2. Identification, Physicochemical Properties, and Subcellular Localization Prediction Analysis of CsKCS Genes

To analyze the physicochemical properties of the screened-out 22 genes, ExPASy Proteomics Server (http://expasy.org/, accessed on 17 January 2023) was used. Table 2 enlists the characteristic properties of *CsKCS* family genes. It was found that the length of *CsKCS* genes varied between 435–930 bp, while the molecular weight of amino acids in the tea tree *KCS* gene family was large (49.23–104.41 Kda). The aliphatic index ranged from 84.36–104.41. Six tea *KCS* proteins were hydrophilic, whereas the others were hydrophobic. The protein encoded by *CsKCS5* exhibited the smallest isoelectric point (pI: 6.17), while the highest pI (9.38) was shown by the protein encoded by *CsKCS19*. Nevertheless, the majority of the *CsKCS* proteins (90.91%) exhibited pI greater than 7.0, indicating the basic nature of proteins.

To predict the subcellular location of tea *KCS* gene family members, software tools such as Softberry (http://linux1.softberry.com/, accessed on 17 January 2023) and WoLF PSORT (https://wolfpsort.hgc.jp/, accessed on 17 January 2023) were employed. The findings revealed that most of the *CsKCS* proteins (90.91%) were localized to the cytoplasm, among which *CsKCS5*, *CsKCS18*, and *CsKCS22* were also localized in chloroplasts. The *CsKCS2* was located within the plasma membrane, while *CsKCS4* was localized to chloroplasts. It indicated that *KCS* proteins accumulate and function mainly within these organelles.

### 2.3. Phylogenetic and Collinearity Analysis of KCS Genes

**Figure 3:**
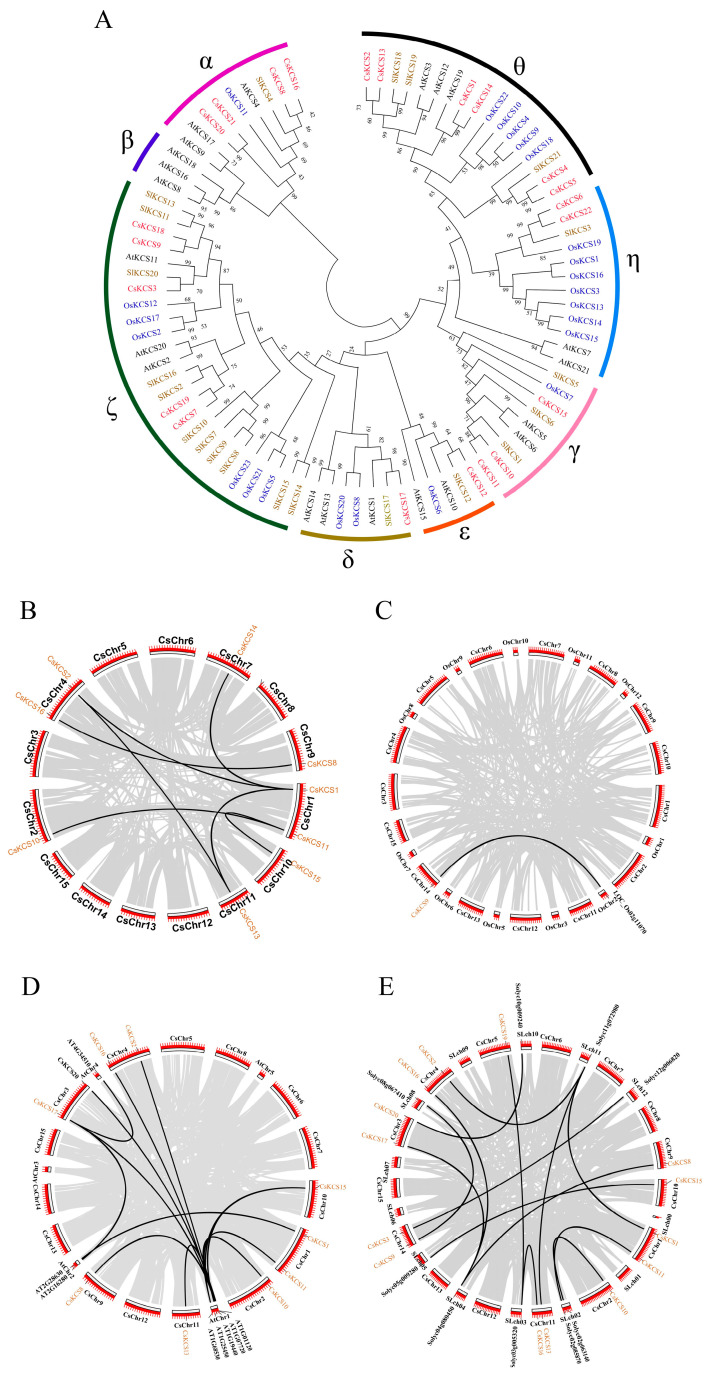
Evolutionary links of the *KCS* gene family in different plants. (**A**) Phylogenetic tree of *KCS* gene family in tea (*Camellia sinensis*), rice (*Oryza sativa*), Arabidopsis (*Arabidopsis thaliana*), and tomato (*Solanum lycopersocum*). The phylogenetic tree was created by aligning full-length protein sequences with MUSCLE, and then using the maximum-likelihood tree method in the MEGA6.0 software The different colors of the outer ring indicate the different groups. *CsKCS* family members are marked in red. (**B**–**E**) Collinearity analysis of *CsKCSs*, (**B**) *CsKCSs* and *CsKCSs*, (**C**) *CsKCSs* and *OsKCSs*, (**D**) *CsKCSs* and *AtKCSs*, (**E**) *CsKCSs* and *SlKCSs*. Black lines indicate the *CsKCS* gene duplication, while gray lines indicate the whole genome duplication events. Red bars denote each chromosome number.

A total of 87 *KCS* protein sequences were selected for sequence alignment to analyze the evolutionary relationship between the sequences. These included 21 Arabidopsis *KCS*, 22 tea plants *KCS*, 23 rice *KCS*, and 21 tomato *KCS* sequences. The adjacency method (Bootstrap = 1000) in the MEGA6.0 (version 6.0, Mega Limited, Auckland, New Zealand) was then used to construct a phylogenetic tree. The *KCS* proteins were divided according to the number and structural characteristics of domains in *Arabidopsis thaliana* [8]. The 87 protein sequences were divided into 8 subfamilies: α, β, γ, δ, ε, ζ, η, and θ. Among them, the θ subfamily was most prevalent, with six *CsKCS* genes, while the ε and δ subfamilies contained only one *CsKCS* gene. There are two *CsKCS* genes in the η subfamily, three *CsKCS* genes in the γ subfamily, four *CsKCS* genes in the α subfamily, and five *CsKCS* genes in the ζ subfamily (Figure 3A).

Tandem duplication and fragment duplication are the two main modes of gene family expansion. To understand the expansion mode of the *CsKCS* gene family of tea plants, the collinearity of the *KCS* gene family of rice, *Arabidopsis*, tomato, and tea plants was analyzed using the TBtools (2022.11 Official R2). It can be seen that a total of 7 homology gene pairs were identified among the 9 *CsKCS* genes of the 22 *CsKCS* family members (Figure 3B). Only one homologous gene pair was identified between the *KCS* genes of the tea plant and rice (Figure 3C). The collinearity analysis of *KCS* presence in genomes of *Arabidopsis thaliana* and tea plant revealed that a total of 12 homologous gene pairs were present amidst10 tea tree *CsKCS* genes and 8 *Arabidopsis AtKCS* genes (Figure 3D). 

The results showed that the *KCS* gene family members of the tea plant and *Arabidopsis thaliana* exhibited substantial collinearity, indicating that many direct homology genes of the *KCS* family of the tea plant and *Arabidopsis thaliana* may have originated from a common ancestor. A total of 14 homologous gene pairs were identified between 14 tea plant *CsKCS* genes and 9 *SlKCS* genes (Figure 3E), suggesting a closer homologous evolutionary relationship between the *KCS* gene families of tomato and tea plants, and these genes may have similar functions.

### 2.4. Analysis of Conservative Protein Motif, Gene Structure, and Chromosomal Location of CsKCS Genes

**Figure 4:**
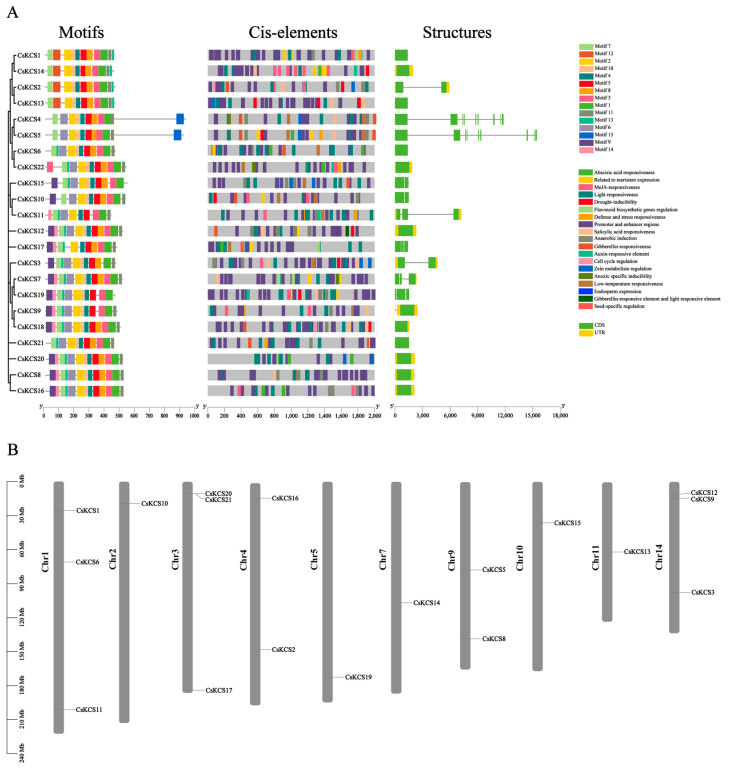
The motif analysis, cis-acting elements analysis, gene structure analysis, and gene location of the *CsKCS* gene. (**A**) *CsKCS* gene protein motifs (right), cis-acting elements (middle), and gene structures (right). Different types of motifs are marked by different color boxes. Various types of cis-elements are represented by different colors. The green bar represents the CDS (coding sequences) and the yellow bar represents Exons (expressed region). (**B**) Mapping of the *CsKCS* gene on 14 chromosomes of the tea plant. The name of the gene is located on the right of each chromosome depending on the location of the *CsKCS* gene.

Motif and gene structural analysis was performed on 22 *CsKCS* genes using online tools (https://meme-suite.org/meme/, accessed on 17 January 2023) and (CFVisual_V2.1, accessed on 17 January 2023) to explore the conserved motif of the *CsKCS* gene family. The PlantCARE (http://bioinformatics.psb.ugent.be/webtools/plantcare/html/) was used to detect cis-control elements in their promoters. Most proteins have the same kind and number of conserved motifs, but there are significant variances, and the distribution of these conserved motifs may reveal different functions of these proteins. All *CsKCS* proteins have more than 10 conserved motifs, with the highest being 13. Motif 1, Motif 2, Motif 4, Motif 5, and Motif 7 are present in each CsKCS sequence. Though motif distribution patterns differ amongst subgroups, members of the same phylogenetic group exhibit similar patterns. For example, *CsKCS1*, *CsKCS2*, *CsKCS13,* and *CsKCS14* have the same number and type of conservative motifs, containing 11 conserved motifs. Similarly, *CsKCS4* and *CsKCS5* have the same number and type of conserved motifs, containing 11 conserved motifs. The number and type of conservative motifs of *CsKCS8*, *CsKCS16,* and *CsKCS20* were also the same, including 14 conservative motifs (Figure 4A).

The mechanisms by which genes respond to different plant hormones and abiotic stresses can be comprehended by promoter analysis. The cis-acting elements, located in the promoter region upstream of the gene, bind to transcription factors, regulate gene transcription, and respond to different environmental factors. Using evolutionary relationships and 2 kb region files upstream of the starting codon, the response elements of *CsKCS* were identified. Most promoters from members of the *CsKCS* gene family contain response elements including light, promoter and enhancer regions, anaerobic induction, low temperature, SA (salicylic acid), MeJA (methyl jasmonate), GA (gibberellin), and others. Since these cis-acting elements are involved in the hormonal response, stress induction, and defense response, it suggests that *KCS* proteins are crucial for hormonal response and stress induction in plants (Figure 4A).

To identify variations in *CsKCS* gene structure between groups, the exons in the *CsKCS* gene sequence were analyzed. The *CsKCSs* contain 0 to 3 exons (Figure 4A). Among them, 10 *CsKCS* genes have no exons, including *CsKCS1*, *CsKCS3*, *CsKCS4*, *CsKCS5*, *CsKCS6*, *CsKCS9*, *CsKCS11*, *CsKCS13*, *CsKCS19*, and *CsKCS20*. The *CsKCS17* and *CsKCS21* have one exon each, whereas *CsKCS7* has three exons. The remaining nine *CsKCS* contain two exons, of which *CsKCS2* and *CsKCS14* belong to the θ subfamily of the *CsKCS* evolutionary tree, *CsKCS10* and *CsKCS15* belong to the γ subfamilies, and *CsKCS8* and *CsKCS16* belong to the α subfamily. These findings point to a variable genetic organization within the same subfamily, suggesting the diversification of gene families.

The distribution of gene families on chromosomes is directly related to the correlation and importance of chromosomes participating in gene family members’ expression and plant growth and development. The TBtools software Gene visualize from GTF/GFF function was used to visually map the distribution of *CsKCS* family members on chromosomes using data from the tea plant genomes gff3 format files. It was found that 18 *CsKCS* genes were unevenly distributed on chromosomes 1, 2, 3, 4, 5, 7, 9, 10, 11, and 14 (Figure 4B). With three members, chromosomes 1, 3, and 14, having the most *CsKCS,* followed by chromosomes 4 and 9 containing two members, and chromosomes 2, 5, 7, 10, and 1 containing only one member.

### 2.5. Expression Analysis of CsKCS Genes under Different Stresses

**Figure 5:**
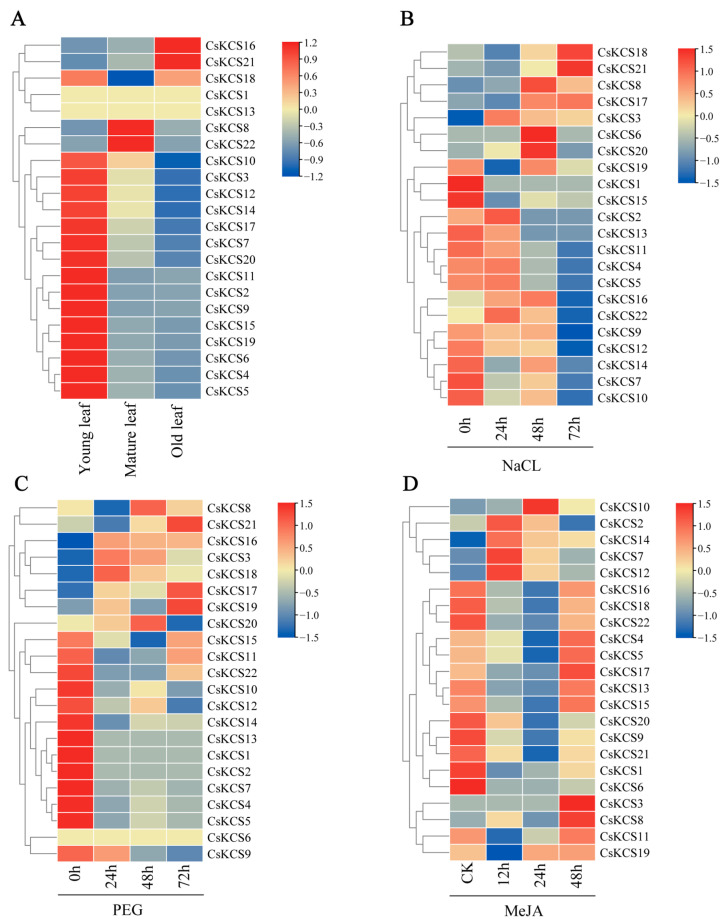
The expression pattern of *CsKCSs.* (**A**) Heat map of the *CsKCSs* expression in tea leaf at different stages of growth, from left to right are young leaf, mature leaf, and old leaf. (**B**) Heat map of the *CsKCSs* expression after NaCl treatment. (**C**) Heat map of the *CsKCSs* expression under drought stress (**D**) Heat map of the *CsKCSs* expression after MeJA treatment. Expression data used are obtained from the tea tree Genome and bioinformatics platform TPIA (Tea Plant Information Archive).

The potential role of 22 *CsKCS* genes in terms of differential expression in stress response was investigated in tea leaves at different growth stages. The transcriptome data and the expression levels of tea leaves at different growth stages were analyzed to comprehend their dynamic responses to three stresses in four stages. The *CsKCSs* expression profiles at various development stages, subjected to methyl jasmonate, drought, and salt stress treatments were obtained. The 22 *CsKCSs* exhibited different expression patterns in tea leaves at different growth stages. Most of the *CsKCSs* were highly expressed in young leaves, except for *CsKCS8* and *CsKCS22*, whose expression was predominant in mature leaves. Similarly, the majority of the *CsKCSs* were less expressed in old leaves, except for *CsKCS16* and *CsKCS21,* which were highly expressed in old leaves (Figure 5A). Only *CsKCS18* was found to be significantly expressed in both young and old leaves, suggesting that it may play an important role in the formation of tea cuticle wax. The variations in expression patterns indicate that distinct *CsKCSs* have different regulatory effects on the leaves of tea plants at different stages.

Under NaCl treatment, four *CsKCS* genes (2, 3, 16, 22) were significantly upregulated at 24 h, four *CsKCS* genes (6, 8, 19, 20) were significantly upregulated at 48 h, and three *CsKCS* genes (17, 18, 21) were significantly upregulated at 72 h. The expression of *CsKCS3* is always upregulated under salt stress (Figure 5B). Under drought stress, the expression of four *CsKCS* genes (3, 16, 17, 18) was consistently upregulated. Nevertheless, the expression of *CsKCS3* and *CsKCS18* were significantly upregulated at 24 h, while at 48 h *CsKCS8* and *CsKCS20* were significantly upregulated, and *CsKCS17*, *CsKCS19,* and *CsKCS21* were significantly upregulated at 72 h (Figure 5C). Under MeJA stress, the expression of four *CsKCS* genes (2, 7, 12, 14) was significantly upregulated at 12 h. Only *CsKCS10* was significantly upregulated at 24 h. At 48 h, the expression of *CsKCS2* was significantly downregulated, while the expression of 12 *CsKCS* genes (3, 4, 5, 8, 11, 13, 15, 16, 17, 18, 19, 22) was upregulated, among which *CsKCS3*, *CsKCS8*, and *CsKCS17* were significantly upregulated (Figure 5D). Almost all *KCS* genes of tea plants were expressed, demonstrating that *CsKCS* genes can respond well to adversity stress, thereby promoting plant growth and development.

### 2.6. Expression Analysis of CsKCSs in Tea Plants with Different Cuticular Wax Content

**Figure 6:**
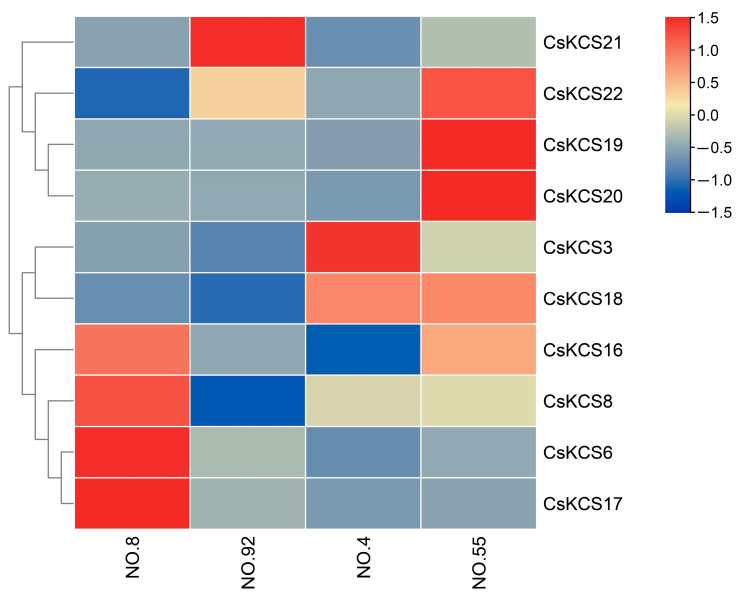
The heat map of expression of selected *CsKCSs* in different Niaowang species (No. 4, 8, 55, 92) with varying leaf wax content.

To further validate the role of *CsKCSs* in the formation of tea epidermal wax, 8 *CsKCS* genes in addition to *CsKCS3* and *CsKCS18* were screened based on the expression of *CsKCSs* in different tissues of tea plants and their response to environmental stress (Figure 6). The transcriptional activity of these 10 *CsKCS* genes in mature leaves of No. 4, No. 8, No. 55, and No. 92 tea plants (Niaowang species) was analyzed by qRT-PCR (Table S3). The expression levels of *CsKCS3* and *CsKCS18* genes were higher in No. 4 and No. 55 Niaowang species with higher wax content in tea cuticle, followed by No. 8 Niaowang species with medium wax content. The expression level was lowest in the No. 92 Niaowang species, which has the least wax content. These findings verified that *CsKCS3* and *CsKCS18* played an important role in the synthesis of tea cuticle wax.

### 2.7. VIGS of CsKCS3 and CsKCS18 in Tea Plants

In this study, the expression levels of *CsKCS3* and *CsKCS18* in tea leaves were downregulated by the TRV (tobacco rattle virus) based VIGS system to study the functions of *CsKCS3* and *CsKCS18* [32].

**Figure 7:**
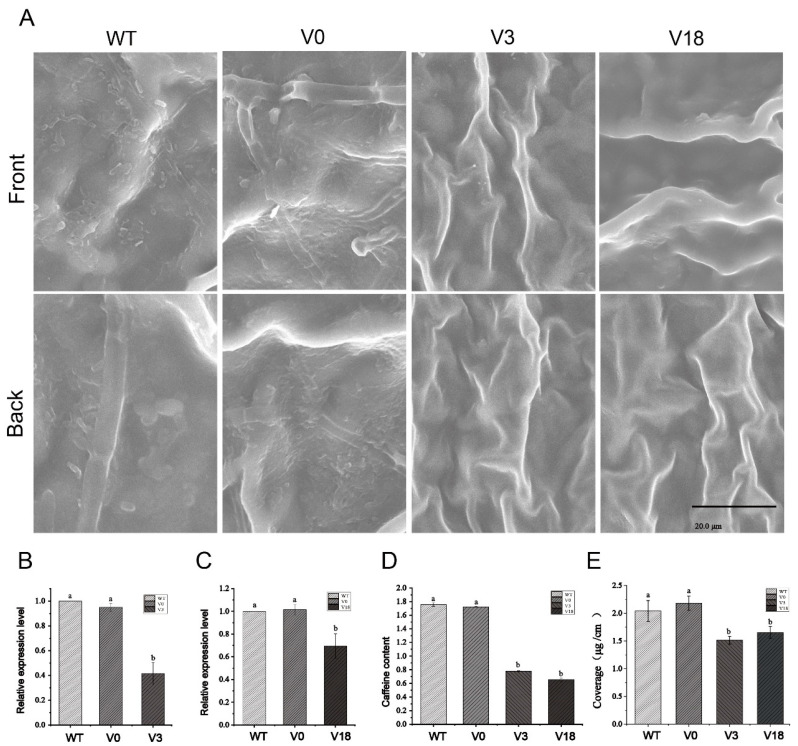
Silencing of *CsKCS3* and *CsKCS18* in a leaf of tea plant using the VIGS system. (**A**) The phenotype of wax crystals on the leaf surface of the tea plant was visualized by SEM. WT, wild type; V0, tea leaf cuttings infected by pTRV1+pTRV2 *Agrobacterium*; V3, tea leaf cuttings infected by pTRV1+pTRV2-*CsKCS3 Agrobacterium*; V18, tea leaf cuttings infected by pTRV1+pTRV2-*CsKCS18 Agrobacterium*. The bar indicated 20.0 µm. (**B**,**C**) The relative expression level of *CsKCS3* (**B**) and *CsKCS18* (**C**) in WT, V0, V3, and V18. (**D**) The caffeine content of WT, V0, V3, and V18. (**E**) Cuticle wax content of WT, V0, V3, and V18. Three technical and biological replicates were used for each data point. Data were presented as means ± Sd (*N* = 3). Different letter in histograms denotes significant difference (*p* < 0.05).

Cuttings infected with pTRV1+pTRV2-*CsKCS3* and pTRV1+pTRV2-*CsKCS18* grew new yellowish leaves after 15 days and were named pTRV2-*CsKCS3* and pTRV2-*CsKCS18*. The newly developed leaves of *Agrobacterium*-treated pTRV1+pTRV2 cuttings and wild-type cuttings were normal green. The tea leaf cuticular wax was observed by SEM, and it was found that the wax quality of the leaf surface with silenced genes was significantly reduced (Figure 7A). The qRT-PCR analysis showed a significant decrease in the expression levels of *CsKCS3* and *CsKCS18* in silenced leaves, indicating that these two genes were silenced (Figure 7B,C). Caffeine accounts for more than 80% of the total tea leaf cuticle wax [30]. Hence, the caffeine content was determined, and it was found that the caffeine content of silenced tea tree leaves was significantly reduced (Figure 7D). The content of cuticular wax was determined, and it was found that the content of cuticular wax in silent tea leaves decreased significantly (Figure 7E, Table S4). The findings validated that *CsKCS3* and *CsKCS18* were positive regulators of tea leaf cuticle wax accumulation.

## 3. Discussion

Extreme weather conditions such as heat waves, droughts, floods, and cold spells have become a major agronomic concern, as global climate warming and environmental stressors accelerate. Leaf stress tolerance is positively associated with leaf cuticular waxes, which form an important protective barrier in the long-term ecological adaptation against harsh environments and biotic and abiotic stresses [33]. Much study has been conducted in recent years on the role of plant waxes in plant resistance to adversity, and the functions of many genes involved in wax synthesis including *CERs* [34], *LACSs* [35], and *KCRs* [36] among others have been verified. Therein, *KCS* is the key enzyme in the synthesis of VLCFAs, the precursors of waxes, which determines the specificity of the tissue and substrate [12]. The overexpression of *CqKCS2B.1* increased the content of C_22_–C_24_ chain length VLCFAs, suggesting that *CqKCS2B.1* plays an important role in the extension of VLCFA [37]. The deletion of the *AtKCS2* inhibits acyl-CoA chain extension, proving KCS is necessary for cuticle wax formation [38]. The simultaneous mutations in *AtKCS5* and *AtKCS6* block drought-induced wax production, suggesting that reactions catalyzed by *KCS* play a key role in wax biosynthesis under drought conditions [39].

The decrease in total cuticular wax content is often accompanied by a decrease in wax crystal density [40,41,42,43]. Previous studies have shown that cuticular wax accumulation is related to the permeability of plant epidermis. Cuticular wax accumulation decreases, chlorophyll leaching rate increases, cuticular wax accumulation increases, and chlorophyll leaching rate decreases [40,41,42,43]. Combined with the coverage density of cuticular wax crystals and chlorophyll leaching rate, it was speculated that the cuticular wax content of leaves of No. 4 and No. 55 Niaowang species was the highest, followed by that of No. 8 Niaowang species, and that of No. 92 Niaowang species was the lowest.

In this study, the transcriptome data of four tea cultivars with substantial variations in cuticular wax content were analyzed. Compared with tea cultivars with low cuticular wax content, *CsKCS3,* and *CsKCS18* were among the top 23 upregulated genes with the highest expression level in tea cultivars with high cuticular wax content, indicating the likelihood of *CsKCSs* involvement in the formation of tea cuticular wax. Although the *KCS* gene family has been linked to plant cuticular wax formation in other species, its role in cuticular wax formation in tea leaves remains unclear. Therefore, it is necessary to further analyze the characteristics of the *CsKCS* gene family to reveal its regulatory role in the synthesis of cuticle wax in tea leaves.

From the tea genome database, 22 *CsKCS* genes containing 2 complete domains like other *KCS* genes were identified. Multi-species phylogenetic analysis revealed that the number of *KCS* found in the genomes of different plant species varied in each group. Except for the β group, *KCS* in the other seven groups was considered to be conserved in all four species, because each of the seven groups comprised *KCS* from all test species (Figure 3A). *KCS* gene family members have traditionally been reported to respond to abiotic and biotic stresses, via the identified *CsKCSs* cis-elements. Further, there were many environmental response elements and hormone response elements such as MeJA-responsiveness, drought-inducibility, and low-temperature responsiveness. The analysis of transcriptomic results from MeJA, NaCl, and drought treatment revealed that *CsKCSs* responded to induction with varying expression titers at different response times. The expression of *CsKCSs* in different stages (young, mature, old) of tea leaves confirmed its regulatory role in all growth phases (Figure 5A). Notably, all *CsKCSs* possess light-responsive cis-acting elements, implying that *CsKCSs* can also be regulated by light.

Previous research has shown that *KCS* is the first rate-limiting enzyme in the synthesis of VLCFAs, and the content of VLCFAs directly affects the cuticular waxes of plants [44]. The expression levels of these 10 *CsKCS* genes in four tea cultivars with different cuticular wax content were analyzed by qRT-PCR. The results showed that the higher the cuticular wax content of tea leaves, the higher the expression levels of *CsKCS3* and *CsKCS18* (Figure 6).

To further verify the positive regulatory role of *CsKCS3* and *CsKCS18* in the formation of cuticular wax, their gene expression was silenced in tea plants by a newly developed VIGS strategy. The silencing of *CsKCS3* and *CsKCS18* resulted in the inhibition of gene expression and the reduction of cuticular wax in tea leaves (Figure 7A), which proved that *CsKCS3* and *CsKCS18* are necessary for the formation of cuticular wax in tea leaves. Meanwhile, the caffeine content of leaves was significantly reduced [30].

Overexpression of *KCS* in wheat significantly increased the resistance to the adversity of wheat [45]. In barley, the mutation of the *KCS1* gene affected the wax structure of the stratum corneum, thereby reducing the resistance to water and powdery mildew of cuticles [46]. The heterologous expression of navel orange *CsKCS6* in Arabidopsis significantly increased the number of VLCFAs in stem and leaf wax and enhanced the tolerance of transgenic Arabidopsis plants to abiotic stress [47]. Under field conditions, severe drought reduces caffeine content in most tea varieties [48]. Drought stress inhibited the expression of caffeine biosynthesis-related genes such as *CsIMPDH*, *CsSAMS*, *CsMXMT*, and *CsTCS*, thereby reducing the accumulation of caffeine in tea leaves [26]. Caffeine is the major component in tea leaf cuticular waxes, accounting for more than 80% of total cuticular wax [30]. The decrease in cuticular wax content may be due to the decrease in VLCFAs and caffeine content caused by the silencing of CsKCS3/CsKCS18.

When *CsKCS3* was silenced but not *CsKCS18*, the leaves of the silenced plants were not covered by cuticle wax crystals, and the caffeine content was substantially reduced. Similarly, when *CsKCS18* was silenced and not *CsKCS3*, the leaves of the silenced plants were not covered by cuticle wax crystals, and the caffeine content also decreased significantly (Figure 7). After CsKCS3/CsKCS18 silencing, the cuticular wax content of tea leaves decreased significantly (Figure 7E). It indicated that *CsKCS3* and *CsKCS18* may need to work together for the synthesis of cuticular wax in tea leaves. These findings imply that *CsKCS3* and *CsKCS18* regulate the synthesis of cuticular wax in tea leaves and also govern the synthesis of caffeine in tea cuticles.

## 4. Material and Methods

### 4.1. Plant Material

The tea cultivar ‘FudingDabai’ was planted under standard field conditions in the experimental field of Guizhou Academy of Agricultural Sciences (latitude 26°11′ N, longitude 106°27′ E, and altitude 1185 m above mean sea level, Guiyang, China). The mature leaves of 108 tea plants (*Camellia sinensis* ‘Guiding Niaowangzhong’) were obtained from the resource garden of Guiding Yunwu Town (latitude 26°17′ N, longitude 107°03′ E, and altitude 1200 m above mean sea level, Guiding, Guizhou, China) when the new shoots of different varieties of the same age grew slightly to a bud with seven leaves, the seventh leaves were collected for analysis

### 4.2. Scanning Electron Microscopy for Observing Tea Leaf Cuticular Wax

Tea leaves were sliced (1 cm × 1 cm) and soaked in a glutaraldehyde-fixed solution (Beijing Solarbio Science & Technology Co., Ltd., Beijing, China) overnight. After gradient dehydration with 30%, 50%, 70%, and 90% ethanol (Sangon Biotech (Shanghai) Co., Ltd., Shanghai, China), followed by freeze drying and gold spraying, an S-3400N scanning electron microscope (Hitachi Limited Co., Ltd., Tokyo, Japan) was used to observe tea leaf cuticular wax.

### 4.3. Determination of Chlorophyll Extraction Rate

The mature leaves of No. 4, No. 8, No. 55, and No. 92 Niaowang species were cut into small pieces about 2 cm long, and 0.2 g of the sheared leaves were placed in a volumetric flask containing 50 mL of 80% ethanol (Sangon Biotech (Shanghai) Co., Ltd., Shanghai, China) solution. The extraction was performed under dark conditions at room temperature for 9 h, during which it was gently shaken every half hour. Using the Hitachi UH5300 dual-beam spectrophotometer (Hitachi Limited Co., Ltd., Tokyo, Japan), the absorbance values D649 and D665 of the extracts at 649 nm and 665 nm were measured after 1 h, 2 h, 3 h, 4 h, 5 h, 6 h, 6 h, 7 h, 8 h, 9 h, and 24 h. Then, the chlorophyll content C at each time point was calculated according to Formula (1). The extraction amount of the last measurement 24 h was C24, and thus, the extraction percentage of each time point = C/C24 × 100%.

(1) C=6.63×D665+18.08×D649

### 4.4. Differentially Expressed Gene Analysis by Transcriptome Sequencing

Total RNA was extracted from mature leaves of four tea germplasms (No. 4, No. 8, No. 55, No. 92) using the TRIzol Up Plus RNA kit (Beijing Tiangen Biochemical Technology Co., Ltd., Beijing, China). Their quality and concentration were detected by a 1.0% agarose (Sangon Biotech (Shanghai) Co., Ltd., Shanghai, China) gel electrophoresis and nucleic acid concentration detector (Implen (Beijing) International Trading Co., Ltd., Beijing, China). Three independent biological replicates were used for RNA sequencing. Each sample (3 μg) was used for sequencing library preparation using the NEBNext^®^ Ultra TM RNA library preparation kit (NEB, Ipswich, MA, USA), according to the manufacturer’s instructions. Then the quality of the library was evaluated by Agilent 2100 biological analyzer system. The Illumina NovaSeq 6000 platform was used for subsequent sequencing. Adaptor sequences, empty reads, and low-quality bases (Q < 30) were removed to obtain high-quality clean reads.

The resulting clean reads were subsequently used for transcriptome de novo assembly by mapping to the tea plant reference genome (http://tpia.teaplant.org/, accessed on 15 December 2022) [49,50]. Fragments Per Kilobase of transcript per Million (FPKM) of each gene and the read counts of the value of each transcript (protein_coding) were calculated using Bowtie2 and eXpress. The differential expression of genes among four tea germplasms was analyzed by DESeq (2012) R package. The FPKM values between four cultivars were compared by a threshold of FDR < 0.001 and |log2ratio| > 1 to investigate differentially expressed genes.

### 4.5. Extraction and Measurement of Cuticle Wax of Tea Leaves

Before extracting cuticular wax from tea leaves, the surface area of the sample was measured using ImageJ (V1.8.0.112 Official Version). Tea leaves were placed in a 50 mL beaker and extracted with 20 mL chloroform for 30 s at room temperature. After filtration with filter paper, as described above, the tea leaves were re-extracted twice. After the extraction solution was mixed, 0.01 mg of C_24_ alkane (Merck Chemicals (Shanghai) Co., Ltd., Shanghai, China) was added as an internal standard. The mixed solution was transferred to a 2 mL sample bottle and dried with nitrogen. Then, 100 μL pyridine (Shanghai Aladdin Biochemical Technology Co., Ltd., Shanghai, China) and 100 μL BSTFA (Shanghai Aladdin Biochemical Technology Co., Ltd., Shanghai, China) were added to redissolve the contents. The resulting extract was heated at 70 °C for 1 h, and chloroform (0.5 mL) was added to re-dissolve the contents dried with nitrogen. The Cuticular wax content is measured using a GC-MS equipped with a DB-5 column (0.25 mm × 30 m, 0.25 μm). The carrier gas (helium) flow rate was set to 1 mL/min. The filtered sample (1 μL) was injected into the GC-MS system for analysis. The inlet temperature was set at 270 °C. The initial oven temperature of 70 °C was maintained for 2 min, increased to 200 °C at a rate of 15 °C/min, maintained for 2 min, increased to 290 °C at a rate of 4 °C/min, maintained for 2 min, and finally increased to 300 °C at a rate of 2 °C/min, which was maintained for the next 10 min. The MS transfer ion source temperature was 320 °C. The electron ionization was carried out at 70 eV. Wax constituents were identified using the NIST2017 database based on the mass spectra. The wax chemical content was relatively quantified by determining the GC-MS peak areas of the wax compound compared to that of the tetracosane (C24) served as internal standards [29].

### 4.6. Bioinformatics Analysis of the CsKCS Gene Family

The tea genome data and proteome sequences were downloaded from the tea tree Genome and bioinformatics platform TPIA (http://tpia.teaplant.org/, accessed on 16 January 2023) database. The C-terminal domain of 3-oxy-(acyl-carrier-protein (ACP)) synthase III (ACP_syn_III_C, Pfam: PF08541) and FAE1/Type III polyketosynthase-like protein domain (FAE1_CUT1_RppA, Pfam: PF08392) were accessed from the Hidden Markov model (HMM) spectrum (http://pfam.xfam.org/, accessed on 16 January 2023) [51]. The *KCS* conservative structure domain (FAE1_CUT1_RppA and ACP_syn_III_C) of tea plant protein candidate sequences (e < 10^−10^) was accessed using the HMMER (https://www.ebi.ac.uk/Tools/HMMER, accessed on 16 January 2023) online retrieval tool. The candidate sequences were submitted to NCBI conservative domains (https://www.ncbi.nlm.nih.gov/structrue/CDD/WRPSB.Cgi, accessed on 16 January 2023) [52] after being pruned of their short and redundant parts. SMART (http://smart.embl-heidelberg.de/, accessed on 16 January 2023) [53] database accession was used to reverify that the *KCS* domain is included. Further, incomplete reading frames, short sequences, and redundant sequences were manually removed, and 22 *KCS* genes in the tea tree were finally obtained and named according to their positions in chromosomes and their homology relationships.

Protein isoelectric point, molecular weight, instability index, aliphatic index, etc. were predicted using the ExPASy Proteomics Server (http://expasy.org/, accessed on 17 January 2023). Softberry (http://linux1.softberry.com/, accessed on 17 January 2023) and WoLF PSORT (https://wolfpsort.hgc.jp/, accessed on 17 January 2023) were used to predict the subcellular localization of *KCS* proteins [54].

The corresponding amino acid sequences were downloaded from Phytozome (https://phytozome.jgi.doe.gov/pz/portal.html, accessed on 17 January 2023), based on the *CsKCS* genes identified in rice, Arabidopsis, and tomato. After amino acid multiple sequence alignments by ClustalW, the phylogenetic tree was constructed by the neighbor-joining method (Bootstrap = 1000) in MEGA6.0 software.

To predict the cis-acting elements in the promoter region, the 2000 bp sequence upstream of the start codon of 22 *CsKCS* genes was submitted to the analysis website PlantCARE (http://bioinformatics.psb.ugent.be/webtools/plantcare/html/, accessed on 17 January 2023).

Motif and gene structure analysis of 22 *CsKCS* genes was performed using online tools (https://meme-suite.org/meme/ and CFVisual_V2.1, accessed on 17 January 2023).

The gene sequences and chromosome files of rice, Arabidopsis, and tomato were obtained from the Ensembl Plants website (http://plants.ensembl.org/index.html, accessed on 18 January 2023). The collinearity of the *KCS* gene family of rice, Arabidopsis, tomato, and tea was analyzed on TBtools (2022.11 Official R2) by using tea gene sequences and chromosome files.

The TBtools (2022.11 Official R2) Gene visualize from GTF/GFF function was used for visual mapping, using the distribution information of *CsKCS* family members on chromosomes in the gff3 format file of the tea plant genome.

Based on the number of *CsKCS* genes identified in tea plants, their transcriptome data (TPM values) in different tissues of tea plants (including tender leaves, mature leaves, and old leaves), salt stress, PEG-induced drought stress, and MeJA treatment were downloaded on the TPIA (https://tpia.teaplant.org, accessed on 24 January 2023) database. The results were used to construct heat maps using TBtools software.

### 4.7. RNA Extraction and qRT-PCR Analysis

Based on the *CsKCS* gene sequences screened from transcriptome data, qRT-PCR (quantitative reverse transcription PCR) primers (Table S5) were designed online using IDT (https://sg.idtdna.com/Pages, accessed on 25 January 2023). The primer sequence was synthesized by Beijing Qingke Biotechnology Co., Ltd. The total RNA was extracted using a TRIzol Up Plus RNA Kit (TianGen Biochemical Technology Co., Ltd., Beijing, China), and its quality and concentration were detected by a 1.0% agarose (Sangon Biotech (Shanghai) Co., Ltd., Shanghai, China) gel electrophoresis and nucleic acid concentration detector (Implen (Beijing) International Trading Co., Ltd., Beijing, China). The RNA was reverse transcribed into cDNA by using the Tiangen Fastking gDNA Dispelling RT SuperMix kit (Beijing Solarbio Science & Technology Co., Ltd., Beijing, China). A real-time qRT-PCR assay was performed on a Bio-Rad CFX Connect^TM^ real-time quantitative PCR instrument (Bio-Rad, Hercules, CA, USA). The qRT-PCR reaction system consisted of 10 μL SYBR Green qPCR Mix, 0.4 μL upstream and downstream primers, 1.5 μL cDNA template, and ddH_2_O to 20 μL. Reaction procedure: 95 °C for 3 min; 40 cycles of 95 °C for 10 s and 60 °C for 20 s; 72 °C for 30 s, with *CsGAPDH* as the reference gene. The 2^−ΔCt^ algorithm was used to calculate the gene expression [55], and the heat map was drawn by TBtools software.

### 4.8. Virus-Induced Gene Silencing of CsKCS3 and CsKCS18 in Tea Plants

For VIGS, the 330 bp *CsKCS3* fragment and the 360 bp *CsKCS18* fragment were assembled into the pTRV2 virus vector to construct the pTRV2-*CsKCS3* vector and pTRV2-*CsKCS18* vector. Then, pTRV1, pTRV2, pTRV2-*CsKCS3,* and pTRV2-*CsKCS18* were transformed into *Agrobacterium tumefaciens* GV3101 strain, respectively. After cultivation and resuspension, the *Agrobacterium* solution carrying pTRV1 was combined in a 1:1 (v:v) ratio with the *Agrobacterium* solution carrying pTRV2 or pTRV2 recombinant vector. The tea cuttings were cut to 20 cm with scissors, and a mature leaf was kept. The tea cuttings were then placed in a Buchner flask containing the above bacterial liquids, respectively mixed, for vacuum infiltration. It was stored in darkness for 3 days and then grown in a 16 h/8 h light/dark cycle at 25 °C in a greenhouse [35].

### 4.9. Determination of Caffeine Content

The caffeine content in leaves of *CsKCS3* and *CsKCS18* silent, wild-type, and non-silent plants was determined by using high-performance liquid chromatography (HPLC) (Hitachi Limited Co., Ltd., Tokyo, Japan). A total of 1.0 g of ground tea samples were weighed on a scale accurate to 0.0001 g and placed in a 500 mL flask. In a boiling water bath, a total of 4.5 g of magnesium oxide (Beijing Solarbio Science & Technology Co., Ltd., Beijing, China), respective sample, and 300 mL of ultrapure water were boiled and extracted for 20 min, with shaking every 5 min. The liquid was filtered under heat and reduced pressure, immediately after extraction. The filtrate was transferred to a 500 mL volumetric flask, cooled down, and mixed well with ultrapure water to bring the volume to proportion. Part of the test solution was filtered through a 0.45 µm membrane filter [56]. The HPLC conditions included a detection wavelength of 240 nm; mobile phase of methanol: ultrapure water (3:7, *v*/*v*); flow rate 1 mL/min; column temperature of 40 °C; and an injection volume of 10 µL, all in accordance with ISO 17027:1995 (2013). The relative content of caffeine was calculated by Formula (2).

(2) $$\mathrm{Caffeine}\left(\%\right)=\frac{C_{1}\times V_{1}}{m\times w\times 10^{6}}\times 100\%$$

### 4.10. Data Analysis

For each measurement, at least three biological replicates were conducted. Data were processed using Microsoft Excel 2021 (Redmond, Washington, DC, USA). SPSS 26.0 (IBM, Inc., Armonk, NY, USA) was used for one-way analysis of variance. The error lines represent the mean ± standard deviation of data from three independent experiments. Different letters denote a significant difference at *p* < 0.05 and a very significant difference at *p* < 0.01.

## 5. Conclusions

In this work, four tea germplasms with significant differences in leaf cuticle wax content were screened. Based on the transcriptome data and leaf cuticle wax content, two *KCS* family genes that may be related to the synthesis of cuticle wax in tea leaves were extensively investigated. A total of 22 *KCS*-encoding genes were identified from the tea plant, and their physicochemical properties, phylogeny, gene structure, and expression pattern were analyzed, generating new information for the gene family. Silencing *CsKCS3* and *CsKCS18* in tea plants by VIGS strategy inhibited the formation of cuticle wax and caffeine in tea leaves. Overall, this study found a strong link between *CsKCS* and the synthesis of tea leaf cuticular waxes and tea stratum corneum caffeine, which provides a foundation for further study of the metabolic mechanism underlying *CsKCS* function and cuticular wax synthesis.

## Figures and Tables

**Table 1:** Cuticle wax content in leaves of 108 tea germplasms.

Tea Leaf Cuticle Content	Tea Germplasms
High content	No. 4, No. 9, No. 11, No. 18, No. 22, No. 25, No. 28, No. 38, No. 39, No. 43, No. 45, No. 47, No. 55, No. 62, No. 65, No. 77, No. 82, No. 91, No. 96, No. 103, No. 108
Medium content	No. 1, No. 2, No. 5, No. 8, No. 10, No. 13, No. 15, No. 16, No. 17, No. 19, No. 20, No. 21, No. 23, No. 24, No. 26, No. 27, No. 29, No. 30, No. 31, No. 32, No. 34, No. 35, No. 36, No. 37, No. 40, No. 41, No. 44, No. 46, No. 48, No. 49, No. 50, No. 51, No. 52, No. 53, No. 54, No. 57, No. 58, No. 63, No. 66, No. 67, No. 72, No. 73, No. 74, No. 75, No. 76, No. 79, No. 80, No. 81,No. 86, No. 87, No. 90, No. 93, No. 97, No. 98,No. 99, No. 104
Low content	No. 3, No. 6, No. 7, No. 12, No. 14, No. 33, No. 42, No. 56, No. 59, No. 60, No. 61, No. 64, No. 68, No. 69, No. 70, No. 71, No. 78, No. 83, No. 84,No. 85, No. 88, No. 89, No. 92, No. 94, No. 95, No. 100, No. 101, No. 102, No. 105, No. 106, No. 107

**Table 2:** The characteristic properties of *CsKCS* family genes.

Gene Name	Gene ID	Gene Location			Protein Properties						Subcellular Localization
		Chr.No	Start	End	Length (aa)	MW/(Kda)	Pl	Instability Index	Aliphatic Index	Hydrophilicity	
*CsKCS1*	CSS0003658	Chr1	25505510	25506898	462	51.96	7.99	36.88	93.27	−0.029	cytoplasm
*CsKCS2*	CSS0004626	Chr4	146654333	146660247	466	52.46	9.03	38.99	87.4	−0.139	Plasma membrane
*CsKCS3*	CSS0005191	Chr14	97178676	97180094	435	49.23	9.08	40.46	90.99	−0.127	cytoplasm
*CsKCS4*	CSS0010963	Contig763	184894	196709	930	104.41	6.37	44.23	91.37	−0.236	chloroplast
*CsKCS5*	CSS0011525	Chr9	77062346	77077789	913	102.66	6.17	43.54	90.82	−0.244	chloroplast, cytoplasm
*CsKCS6*	CSS0013672	Chr1	70974884	70976278	464	52.48	7.95	42.82	93.66	0.059	cytoplasm
*CsKCS7*	CSS0014846	Contig56	380801	385441	514	58.10	9.18	36.52	96.36	−0.015	cytoplasm
*CsKCS8*	CSS0025444	Chr9	138119554	138121704	518	57.86	9.09	37.45	96.12	−0.008	cytoplasm
*CsKCS9*	CSS0025961	Chr14	14320192	14321730	465	52.65	8.87	44.55	93.7	−0.092	cytoplasm
*CsKCS10*	CSS0029062	Chr2	19083909	19085399	460	52.34	9.31	37.38	97.89	0.018	cytoplasm
*CsKCS11*	CSS0030456	Chr1	201268181	201269674	474	53.85	9.28	38.99	101.18	−0.01	cytoplasm
*CsKCS12*	CSS0031750	Chr14	10648591	10655818	543	61.34	9.13	42.88	84.36	−0.171	cytoplasm
*CsKCS13*	CSS0032609	Chr11	61406575	61407960	461	52.11	8.81	40.58	87.55	−0.193	cytoplasm
*CsKCS14*	CSS0032944	Chr7	106477234	106479183	458	51.65	8.68	35.39	96	−0.063	cytoplasm
*CsKCS15*	CSS0037394	Chr10	36097474	36099342	497	56.15	9.2	45.93	104.41	0.114	cytoplasm
*CsKCS16*	CSS0038600	Chr4	13063749	13065843	517	57.68	9.36	37.8	95.05	−0.046	cytoplasm
*CsKCS17*	CSS0038850	Chr3	183897657	183899975	530	59.42	8.94	31.08	92.53	0.013	cytoplasm
*CsKCS18*	CSS0041783	Contig18	419337	421796	511	57.64	9.08	41.02	92.7	−0.031	chloroplast, cytoplasm
*CsKCS19*	CSS0044924	Chr5	172224143	172226480	469	52.83	9.38	33.79	98.96	0.054	cytoplasm
*CsKCS20*	CSS0045193	Chr3	10280927	10282456	509	57.22	9.24	37.75	92.89	−0.087	cytoplasm
*CsKCS21*	CSS0046169	Chr3	10298869	10300437	451	51.11	9.36	34.67	98.18	0.079	cytoplasm
*CsKCS22*	CSS0047402	Chr1	71036309	71037995	536	60.58	8.23	41.76	90.19	−0.067	chloroplast, cytoplasm

## Data Availability

The data presented in this study are available on request from the corresponding author.

## Supplementary Materials

The following supporting information can be downloaded at: https://www.mdpi.com/article/10.3390/foods12102011/s1, Table S1: Cuticular wax content in leaves of No.4, No.8, No.55 and No.92 Niaowang species; Table S2: Niaowang species (No.92, No.8, No.4, No.55) RNA-seq expression data of the first 23 up-regulated genes; Table S3: The expression levels of 10 CsKCS genes; Table S4: Cuticle wax content of WT, V0, V3, and V18; Table S5: 10 *CsKCS* and *CsGAPDH* qRT-PCR primers.
