# Supplementary material for: Genome-Wide Investigation and Functional Analysis Reveal That CsKCS3 and CsKCS18 Are Required for Tea Cuticle Wax Formation
Source: Foods. 2023 May 16;12(10):2011. doi: 10.3390/foods12102011 (PMC10217411; doi:10.3390/foods12102011)
Supplement: Supplementary file 1 [file foods-12-02011-s001.zip › Table S1.pdf]

Table S1 Cuticular wax content in leaves of No.4, No.8, No.55 and No.92 Niaowang species.

Niaowang species	Coverage（μg/cm^2）		
	C1	C2	C3
No.92	5.96	5.64	6.64
No.8	6.50	6.90	6.84
No.4	7.86	8.48	8.66
No.55	8.54	8.24	8.54

C1, C2, and C3 represent biological replicates.
