# Supplementary material for: Genome-Wide Investigation and Functional Analysis Reveal That CsKCS3 and CsKCS18 Are Required for Tea Cuticle Wax Formation
Source: Foods. 2023 May 16;12(10):2011. doi: 10.3390/foods12102011 (PMC10217411; doi:10.3390/foods12102011)
Supplement: Supplementary file 1 [file foods-12-02011-s001.zip › Table S2.pdf]

Table S2 Niaowang species（No.92、No.8、No.4、No.55）RNA-seq expression data of the first 23 up-regulated genes.

Famliy	ID	Niaowang species			
		No.92	No.8	No.4	No.55
P450	CSS0025111	12.18009992	35.5273117	86.22365432	80.4848312
bZIP	CSS0045460	7.331109051	17.68398554	65.22365098	84.94482824
	CSS0041321	11.88982724	36.68052736	69.60697518	74.29942944
NAC	CSS0007957	5.649356885	98.54941837	164.6619835	186.1230524
	CSS0024591	20.96745591	46.04833781	81.98715859	93.99719365
	CSS0030435	47.88972494	89.07942339	164.0892179	157.3855308
SBP	CSS0028528	4.13533356	27.20454491	82.25567038	77.77250415
KCS	CSS0019139	1.200120826	7.74581005	22.70466532	27.42418865
	CSS0036872	9.147244432	27.51752135	88.96633732	81.81514961
B3	CSS0030652	2.549764724	14.41866816	115.6554921	145.1215654
MYB	CSS0016950	0.121726457	1.295714391	73.89167583	65.88397899
AP2	CSS0015911	1.387966851	4.82911696	11.42283182	15.37848272
	CSS0014984	23.74277617	58.78029638	118.3173054	126.4338968
bHLH	CSS0017515	13.89976782	32.3415574	157.8264183	129.0395629
GRAS	CSS0023134	28.60505601	97.22330988	256.8587248	273.5038984
	CSS0005560	15.86279681	44.91529003	133.6132909	117.382075
WRKY	CSS0001109	12.52510036	60.09384307	411.2216069	369.509901
	CSS0016385	17.46386846	147.3803801	409.85527	389.3628475
	CSS0011253	16.30108015	39.07946495	53.00791569	47.59290638
HSP40	CSS0017243	10.05987594	29.82385139	91.70996702	98.18798829
	CSS0011843	12.91277431	38.86222887	112.5568252	166.6644056
	CSS0005191	13.61385661	32.43686576	88.93239062	112.4082839
Dof	CSS0041783	13.63881961	71.64869861	347.9591537	479.6998202
