# Supplementary material for: Genome-Wide Investigation and Functional Analysis Reveal That CsKCS3 and CsKCS18 Are Required for Tea Cuticle Wax Formation
Source: Foods. 2023 May 16;12(10):2011. doi: 10.3390/foods12102011 (PMC10217411; doi:10.3390/foods12102011)
Supplement: Supplementary file 1 [file foods-12-02011-s001.zip › Table S4.pdf]

Table S4 Cuticle wax content of WT, V0, V3, and V18

Sample	coverage（μg/cm^2）		
	C1	C2	C3
WT	1.84	1.99	2.29
V0	2.17	2.03	2.34
V3	1.53	1.59	1.42
V18	1.77	1.68	1.52

C1, C2, and C3 represent biological replicates.WT, wild type; V0, tea leaf cuttings infected by pTRV1+pTRV2 Agrobacterium; V3, tea leaf cuttings infected by pTRV1+pTRV2-CsKCS3 Agrobacterium; V18, tea leaf cuttings infected by pTRV1+pTRV2-CsKCS18 Agrobacterium.
