# Supplementary material for: Genome-Wide Investigation and Functional Analysis Reveal That CsKCS3 and CsKCS18 Are Required for Tea Cuticle Wax Formation
Source: Foods. 2023 May 16;12(10):2011. doi: 10.3390/foods12102011 (PMC10217411; doi:10.3390/foods12102011)
Supplement: Supplementary file 1 [file foods-12-02011-s001.zip › Table S5.pdf]

**Table S5** 10 *CsKCS* and *CsGAPDH* qRT-PCR primers.

| Primer ID | Forward Primer | Reverse Primer | Amplicon |
|---|---|---|---|
| *CsKCS3* | GAGGAGCTGCAATACTCCTATC | GAGTCCTCACGGTATGTACTAATC | 81 |
| *CsKCS6* | ACCAATCTCTCTCCTCTCGTTA | ATCGTCTCGCATGGCATATT | 111 |
| *CsKCS8* | GCAGGATGATGTTGGGAAGA | ACCACCAGAGTAGCAAAGAAC | 147 |
| *CsKCS16* | GCGAGCAACTTCTGTTCTTTG | TCTTCTCCAGCTCATCAATCAC | 147 |
| *CsKCS17* | GGTCTAAGATTGGACCGGATTT | GTAAAGGGCGAAGAGGAAGAG | 111 |
| *CsKCS18* | GGAGACAGAATGTGGCAGATAG | TCCATGGGTTCTTCTCCTTTG | 106 |
| *CsKCS19* | TTTCCTAGCCACTCTCCATTTC | CCTGCCTGTACAGACCTATCTA | 129 |
| *CsKCS20* | CACCTCTTCACTCTCTCTCTACT | ACGGTGACGAGGTTGTATTG | 119 |
| *CsKCS21* | CAACCCAACACCTTCTCTATCT | ACCTGCACTACAACCCATAC | 100 |
| *CsKCS22* | CACACACTCTCTCTCTCTCTCT | AGCTCCATGAGGGTTGAATG | 125 |
| *CsGAPDH* | CCCTCTAAGTCCTCTCTCTCCT | GTTGCTTGGGCAGCCACTACAT | 158 |
